# High‐Efficiency Thermal Battery Regulated by Ultralow Magnetic Fields

**DOI:** 10.1002/smsc.202500498

**Published:** 2025-12-12

**Authors:** Lingli Li, Haoyu Wang, Zhiyu Wang, Dan Huang, Kun Zhang, Bing Li

**Affiliations:** ^1^ Shenyang National Laboratory for Materials Science Institute of Metal Research Chinese Academy of Sciences (IMR, CAS) Shenyang 110016 P. R. China

**Keywords:** barocaloric effects, magnetic control, plastic crystals, solid‐state caloric materials, thermal batteries

## Abstract

Controllable thermal storage has emerged as a central theme in advanced energy management, where external stimuli such as light, stress, and pressure can be exploited to precisely regulate heat release. Yet, realizing efficient and practical deployment requires the development of simpler noncontact actuation methods and the enhancement of heat‐transfer efficiency, both of which remain major challenges. Herein, a magneto‐responsive phase‐change composite is presented by integrating a supercooled plastic crystal, 2‐amino‐2‐methyl‐1,3‐propanediol (AMP), with dispersed NdFeB particles. This design enables noncontact triggering of supercooled phase transitions under ultralow magnetic fields as small as ≈0.04 T. Meanwhile, the dispersed magnetic particles enhance thermal conduction and promote synchronous multipoint crystallization, thereby markedly accelerating heat release. The optimized 20% AMP/NdFeB composite achieves a colossal entropy change of 507.6 J kg^−1^ K^−1^, a corresponding enthalpy change of 181.1 J g^−1^, and a rapid temperature rise of 47.6 K, substantially outperforming leading magnetocaloric systems under far milder field conditions. This work establishes a transformative and generalizable route to noncontact, high‐efficiency, and controllable thermal batteries, paving the way for their practical deployment in advanced energy systems.

## Graphical abstract

A magneto‐responsive composite is reported that, for the first time, achieves a noncontact phase transition with an ultralow magnetic field (≈0.04 T). It exhibits a colossal entropy change of ≈507.6 J kg^−1^ K^−1^, an enthalpy of 181.1 J g^−1^, and a ≈47.6 K temperature rise, alongside enhanced thermal conductivity and heat‐release efficiency, making it promising for thermal batteries.

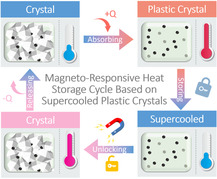

## 1 Introduction

The continuous growth in global energy consumption, coupled with the intermittent and fluctuating nature of renewable energy sources, has posed significant challenges to achieving stable and reliable energy supply.^[^ ^1^, ^2^, ^3^ ^]^ Simultaneously, vast amounts of low‐ and medium‐grade heat are wasted daily across diverse sectors such as industrial manufacturing and transportation, representing a substantial untapped energy resource.^[^ ^4^, ^5^, ^6^ ^]^ Efficiently capturing, storing, and controllably releasing this thermal energy is thus recognized as a key enabling technology for next‐generation energy systems, and a vital pathway toward global carbon neutrality.^[^ ^7^, ^8^ ^]^

Solid‐state phase change materials (SSPCMs) have emerged as promising candidates for thermal energy regulation and redistribution owing to their high energy density, excellent structural stability, and good cyclability.^[^ ^8^, ^9^, ^10^, ^11^ ^]^ They have been widely deployed in solar thermal storage,^[^ ^12^ ^]^ smart grids,^[^ ^13^ ^]^ and electronic thermal management.^[^ ^14^ ^]^ However, conventional SSPCMs are typically triggered passively by ambient temperature changes, limiting the precise control over the timing and rate of heat release.^[^ ^15^, ^16^, ^17^, ^18^, ^19^, ^20^ ^]^ This inherent lack of thermal programmability makes it challenging to adapt to dynamic and complex thermal environments.^[^ ^21^ ^]^ More critically, under storage conditions, these materials are prone to premature heat dissipation upon cooling due to spontaneous phase transitions, impeding long‐term energy retention.

Recently, the concept of field‐controlled thermal storage, using optical/mechanical/magnetic stimuli to modulate heat release, enables highly tunable, intelligent, long‐term, low‐loss thermal batteries.^[^ ^10^ ^]^ For example, azobenzene‐containing polymers^[^ ^22^, ^23^ ^]^ offer rapid visible‐light response, but face challenges due to shallow light penetration and device constraints. Force‐induced systems, such as ceramics (λ‐Sc_*x*_Ti_3−*x*_O_5_)^[^ ^24^ ^]^ and shape memory alloys (TiNi‐based alloys),^[^ ^25^ ^]^ utilize large thermal hysteresis (Δ*T*_hys_) to enable mechanical triggering (≫100 MPa)^[^ ^18^ ^]^ of heat release. Besides, magnetocaloric materials with large Δ*T*_hys_ also show potential for thermal storage and heat pump,^[^ ^26^, ^27^ ^]^ while a key limitation in these materials is their low entropy change (Δ*S* < 100 J kg^−1^ K^−1^), significantly reducing practical energy density and thermal performance. More recently, plastic crystals exhibiting the inverse colossal barocaloric effect (e.g., in NH_4_SCN)^[^ ^28^ ^]^ or a supercooled state^[^ ^29^, ^30^ ^]^ analogous to undercooled liquids have been explored for pressure‐controlled thermal storage. Notably, 2‐amino‐2‐methyl‐1‐propanol (AMP) requires minimal pressure to trigger colossal heat (≈661.8 J kg^−1^ K^−1^) release from its supercooled state, offering exceptional thermal performance beyond traditional phase change materials for long‐term, efficient thermal storage.^[^ ^30^ ^]^

However, although pressure‐regulated plastic crystals have demonstrated the feasibility of constructing pressure‐controlled thermal batteries with objectively superior performance, particularly in terms of large latent heat storage, their practical deployment still faces challenges. These strategies typically rely on contact‐based actuation or specifically engineered structures, which constrain scalability, integration, and rapid responsiveness, especially in compact energy systems.^[^ ^30^, ^31^, ^32^ ^]^ Moreover, efficient thermal management fundamentally depends on heat transfer capability, yet plastic crystals intrinsically suffer from ultralow thermal conductivity because the strong coupling between molecular orientational disorder and low‐frequency lattice vibrations severely suppresses phonon transport. Consequently, overcoming these bottlenecks and developing noncontact, energy‐efficient, and high‐performance strategies for precisely controllable thermal storage remains a pressing challenge.

In this work, we propose and demonstrate a magneto‐responsive thermal battery (AMP/NdFeB) that operates under ultralow magnetic fields. As a quintessential noncontact external stimulus, magnetic fields offer several distinct advantages including remote actuation, strong penetration capability, and compatibility with encapsulated architectures without requiring electrodes or direct interfaces.^[^ ^33^, ^34^ ^]^ These features render magnetic‐field control especially attractive for demanding thermal management scenarios such as aerospace and flexible electronics. Our system achieves effective and reversible heat release (≈507.6 J kg^−1^ K^−1^) under tens of millitesla‐level fields (≈0.04 T), integrated seamlessly within the bulk phase change medium without additional actuators. More importantly, the incorporation of magnetic particles not only enables magnetic‐field responsiveness but also significantly improves the effective thermal conductivity of the composite, thereby overcoming the long‐standing bottleneck of poor heat transfer in organic SSPCMs. This work pioneers a new paradigm for noncontact, energy‐efficient, and controllable thermal storage, opening promising opportunities for next‐generation energy and thermal management technologies.

## 2 Results and Discussion

### 2.1 Overview of the Thermal Battery Regulated by Ultralow Magnetic Fields

**Figure 1:**
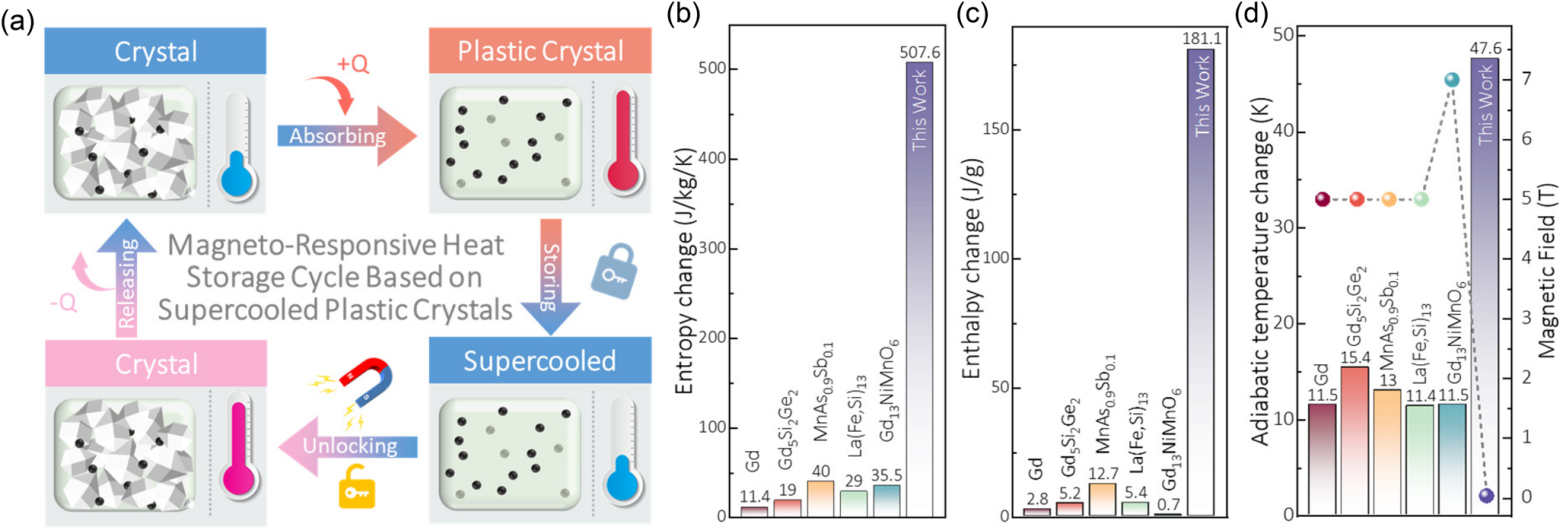
Principle and performance of the magneto‐responsive heat storage cycle based on supercooled plastic crystals. a) Schematic of the heat storage and release cycle in the AMP/NdFeB composite. b–d) Performance benchmarking of the magneto‐responsive supercooled plastic crystals AMP/NdFeB (20%) composite against leading magnetocaloric materials in terms of (b) Δ*S*, (c) Δ*H*, and (d) Δ*T*_ad_ and magnetic field.

To achieve the desired long‐term and field‐controlled thermal storage application, **Figure** **1**a illustrates the schematic of a magneto‐responsive heat storage and release cycle based on supercooled materials. During the endothermic process (+*Q*), the material absorbs high‐grade thermal energy (e.g., from solar or industrial waste heat) to transition from its stable crystalline phase to a high‐temperature disordered phase. Upon cooling, this disordered state is preserved in the supercooled phase, effectively locking the latent heat within the material, even at ambient or sub‐ambient temperatures. Subsequently, for the heat release process (−*Q*), the application of an external magnetic field is designed to trigger crystallization, thereby liberating the stored thermal energy on demand. In this study, we reported this magnetically controlled thermodynamic cycle by creating a composite comprising the supercooled plastic crystal AMP and NdFeB powder.

It's common knowledge that thermal performance of caloric materials is benchmarked by three key parameters, including the entropy change, the adiabatic temperature change (Δ*T*_ad_), and the magnitude of the requisite applied magnetic field. We therefore compared our AMP/NdFeB composite, which functions indirectly by converting field‐induced particle motion into stress, against conventional magnetocaloric materials governed by the direct magnetocaloric effect (Figure 1b–d). The results for the AMP/NdFeB composite (NdFeB is 20% of the total mass ratio) exhibit outstanding performance, achieving a remarkable entropy change (Δ*S*) of ≈507.6 J kg^−1^ K^−1^ (from the heating curve), a corresponding enthalpy change (Δ*H*) of 181.1 J g^−1^, and a large adiabatic temperature change (Δ*T*_ad_) of ≈47.6 K. These values are prominent larger than those of leading magnetocaloric materials including Gd (≈11.4 J kg^−1^ K^−1^, ≈2.8 J g^−1^, ≈11.5 K),^[^ ^35^ ^]^ Gd_5_Si_2_Ge_2_ (≈19 J kg^−1^ K^−1^, ≈5.2 J g^−1^, ≈15.4 K),^[^ ^36^ ^]^ MnAs_0.9_Sb_0.1_ (≈40 J kg^−1^ K^−1^, ≈12.7 J g^−1^, ≈13 K),^[^ ^37^ ^]^ La(FeSi)_13_ (≈29 J kg^−1^ K^−1^, ≈5.4 J g^−1^, ≈11.4 K),^[^ ^38^ ^]^ and Gd_2_NiMnO_6_ (≈35.5 J kg^−1^ K^−1^, ≈0.7 J g^−1^, ≈11.5 K).^[^ ^39^ ^]^ The enthalpy change (Δ*H*) during the phase transition was calculated from Δ*S* using the fundamental thermodynamic relationship Δ*H* = Δ*S*/*T*, where *T* is the phase transition temperature.^[^ ^40^ ^]^ More importantly, this giant caloric effect is triggered by a minute magnetic field change of only ≈0.04 T, sourced from a permanent magnet. This required field is approximately two orders of magnitude lower than the average and reliable magnetic field source (typically > 1 T) essential for conventional magnetocaloric materials.^[^ ^33^, ^35^, ^36^, ^37^, ^39^, ^41^ ^]^ This combination of a giant caloric response under a negligible magnetic stimulus underscores the novelty and high performance of our designed composite system.

### 2.2 Phase Transition under Magnetic Fields

**Figure 2:**
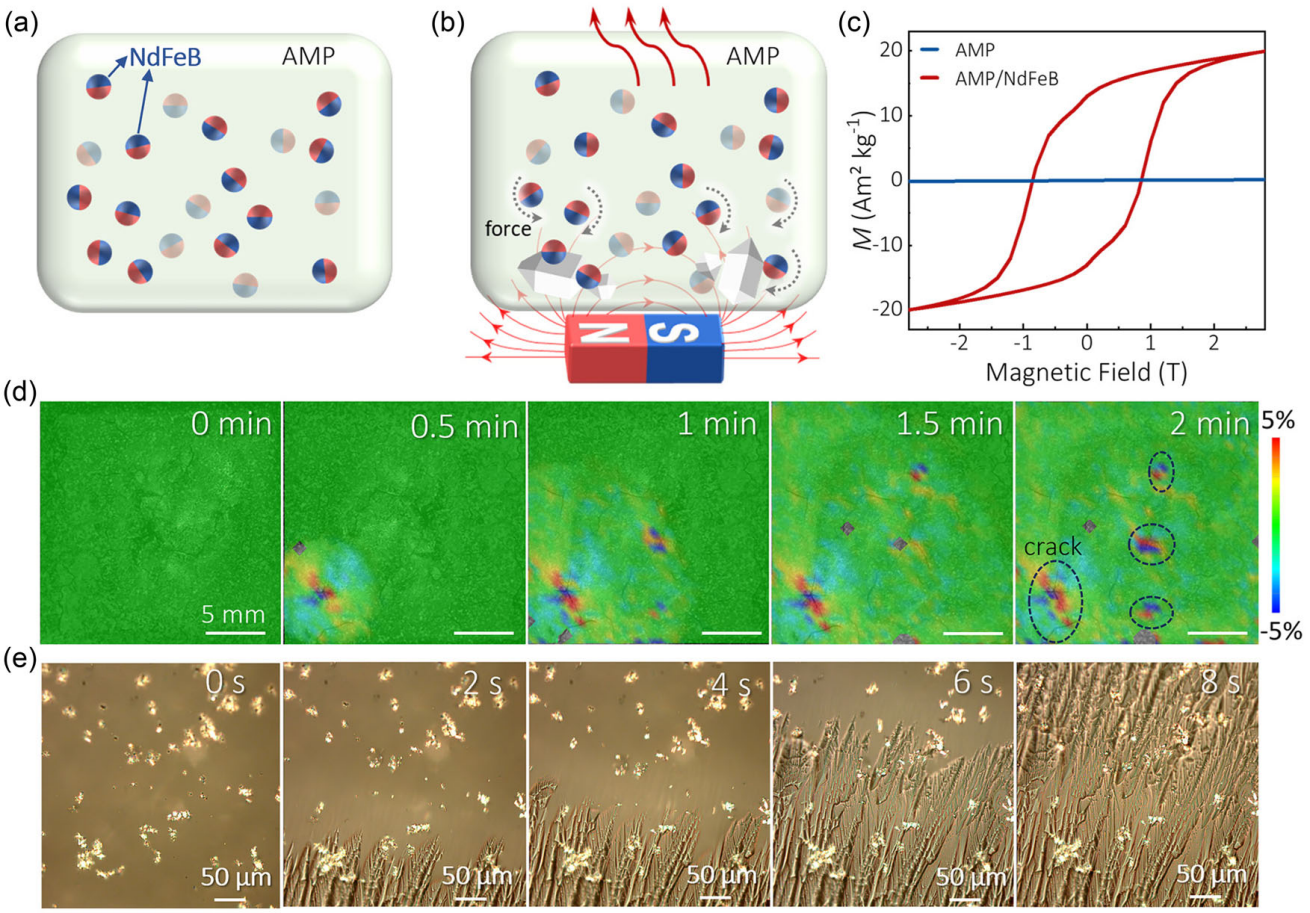
Magnetic field‐induced phase transition schematic diagram and relevant optical photos. a,b) Schematic diagram, c) measured magnetic hysteresis loop of the AMP/NFB with different volume concentrations, d) DIC investigating the strain, and e) optical photos of 20% AMP/NFB during the phase transition.

To investigate the phase change behavior, we prepared pure AMP and AMP/NdFeB composites (NdFeB is 20%, 40%, 60%, and 80% of the total mass ratio, respectively). **Figure** **2**a,b shows the mechanism diagram of magnetically controlled phase transition of AMP/NdFeB at supercooled state. First, AMP with a fixed quality was mechanically mixed with the unmagnetized magnetic NdFeB powder. After heating at ≈110 °C and then cooling down to room or lower temperature, a uniform mixture with transparent supercooled AMP and black NdFeB was obtained. Then, a permanent magnet was placed at a specific position to generate a magnetic field (the magnetic field strength is as low as 0.04 T), which drives NdFeB powder to move or rotate. The movements from powder provide stress and nucleation sites for supercooled AMP transferring to crystal phase. Initiating in regions of heightened magnetic field intensity, the phase transition propagates radially, concurrently releasing huge amounts of heat. Figure 2c shows the magnetic hysteresis (*M*‐*H*) loops of AMP and 20% AMP/NdFeB composite. The results indicate that AMP exhibits weak paramagnetism, while the sample mixed with NdFeB demonstrates strong ferromagnetism, characterized by a remanence of 15 Am^2^ kg^−1^ and a coercivity of ≈0.86 T. It is proved that the composites can respond to magnetic fields.

To quantify the magnetically induced stress in the samples, we employed in situ digital image correlation (DIC) to monitor the concurrent stress and strain evolution during the phase transition of AMP/NdFeB composites in real time, Figure S1a, Supporting Information. As expected, DIC results in Figure S1b, Supporting Information, confirm that the pure AMP sample exhibits almost no detectable strain under magnetic field exposure. Since 20% AMP/NdFeB composites easily undergo magnetically controlled phase transition, a more dilute composite (NdFeB is 10%) was prepared with a rectangular magnet positioned vertically beneath the sample back for DIC measurements to isolate magnetically induced stress prior to phase transition. Figure S1c, Supporting Information, reveals ±0.5% strain exclusively in magnet‐exposed regions, with negligible strain in field‐free zones. Therefore, the composites confer magneto‐responsive functionality on AMP. Furthermore, Figure 2d captures the strain fluctuation during magnetically triggered phase transition, showing nucleation and evolution from a single point. In particular, phase transition‐induced lattice dilation drives crack propagation (>5% strain).

In addition, to distinguish microscopic disparities in crystallization, we employed optical microscopy to track transformation processes, analyzing across gradient weight mixing ratios. Figure 2e and S2, Supporting Information, show the optical microscope photos of the spreading phase transition of composites with different mixing ratios. It reveals that the grain size progressively reduces with increasing the mixing ratio, confirming that magnetic powder provides additional heterogeneous nucleation sites.

### 2.3 Microstructures and Stability of AMP/NdFeB

**Figure 3:**
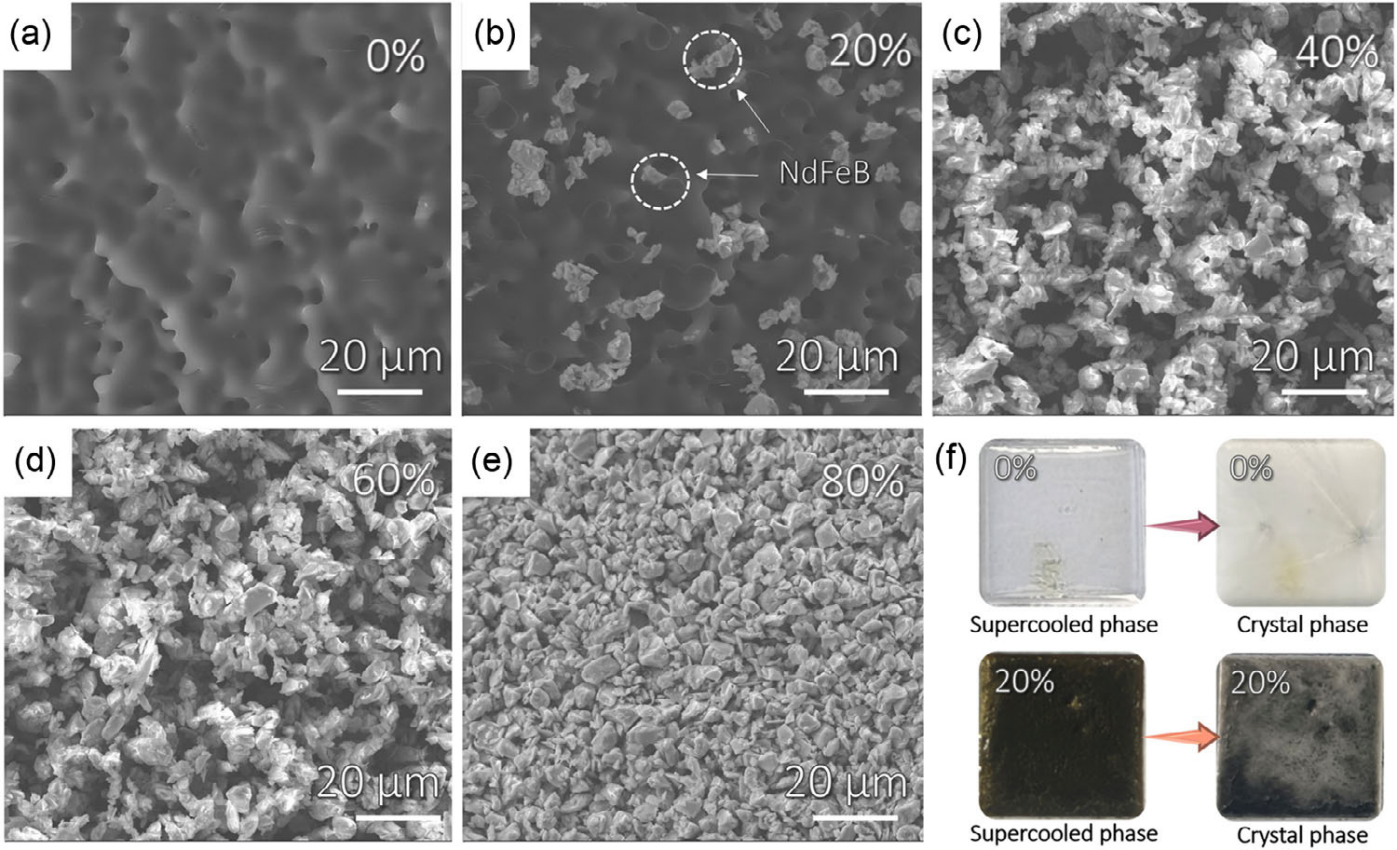
The microstructures of AMP and NdFeB. a–e) SEM images of 0–80% AMP/NdFeB composites; f) the optical photos of AMP and 20% AMP/NdFeB at crystal and supercooled state, respectively.

To elucidate the coexistence mechanism and stability of phases AMP and NdFeB, we characterized the supercooled state of AMP/NdFeB with different ratios by SEM, as shown in **Figure** **3**a–e. 0% AMP/NdFeB shows an amorphous morphology and some holes on the surface, which is quite different from most crystal materials. Besides, NdFeB powder with different mixing ratios can coexist stably with supercooled AMP, with its particle size in the mixed samples ranging between 2 and 5 μm. The optical photos of AMP and 20% AMP/NdFeB suggest that two phases exhibit different colors, while the supercooled and crystal states exhibit transparent and white respectively (Figure 3f). Figure S3, Supporting Information, shows micrographs of the additional samples.

**Figure 4:**
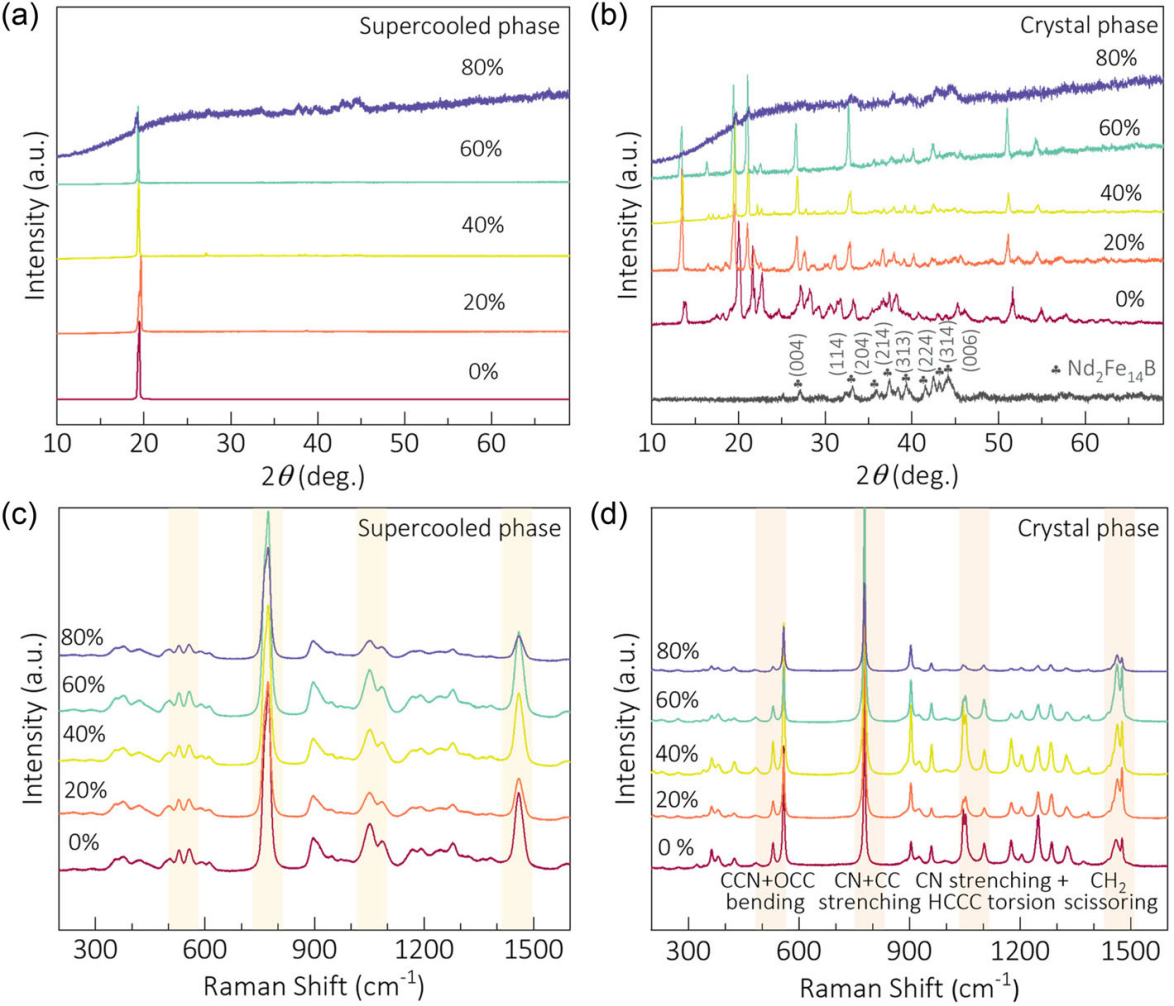
XRD and Raman spectra of different AMP/NdFeB composites. a,b) XRD and c,d) Raman spectra of 5 composites at crystal and supercooled state.

After that, XRD characterization revealed distinct signatures for supercooled and crystalline states. The supercooled state exhibited a characteristic peak at ≈20° across varying compositions, as shown in **Figure** **4**a,b. At 80% mixing ratio, detectable NdFeB signals emerged in two states without additional diffraction peaks. This indicates that the two materials are independent and stable without crystal structure changes, and NdFeB powder did not undergo chemical reaction with AMP. The two phases have also been confirmed optically by using Raman spectroscopy as shown in Figure 4c,d, where typical Raman peak positions (such as 530 and 700 cm^−1^)^[^ ^30^, ^42^ ^]^ show changes across the phase transition. Note these typical peaks mainly reflect the vibration modes of CCN + OCC bending at 530 cm^−1^, CN + CC stretching at 700 cm^−1^, and stretching CN and HCCC torsion between 1100 and 1200 cm^−1^. The Raman spectra are similar for pure AMP and AMP/NdFeB without NdFeB signal peaks, showing the coexistence state of two phases, and the signal intensity decreases with the increase of mixing ratios. Thus, these results further prove that NdFeB can stably coexist with the supercooled state of AMP without chemical reaction.

### 2.4 Heating Performance Evaluation

**Figure 5:**
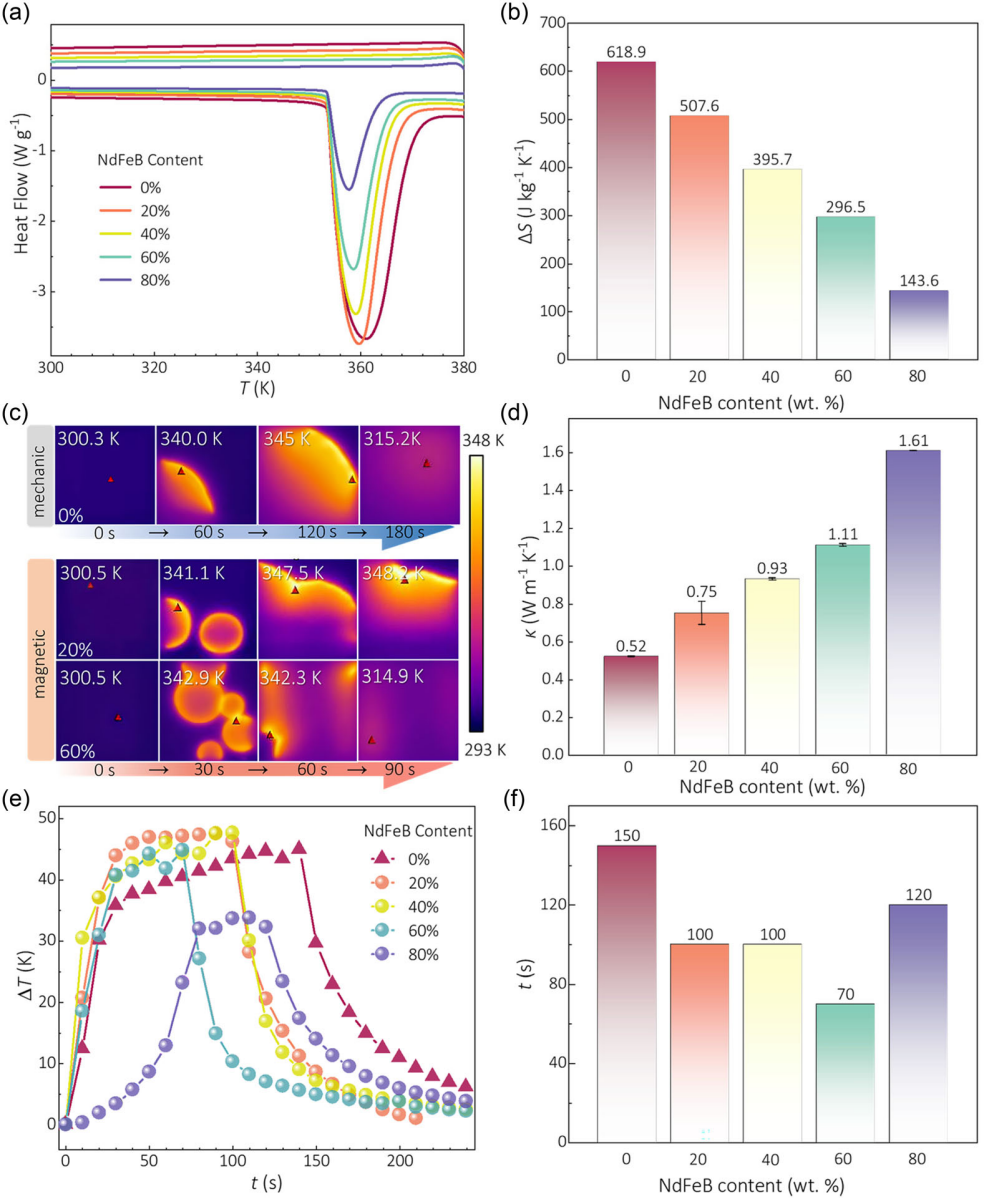
Thermophysical parameters and temperature change induced by magnetic fields and a tip force of AMP/NFB composites. a,b) DSC curves with related entropy, c) infrared thermography pictures exhibiting the heat releasing processes by mechanical and magnetic methods (the red triangle marks the highest‐temperature point in real time), d) the thermal conductivity variation of different AMP/NdFeB composites, e) temperature variation curves of real‐time maximum temperatures in infrared thermography (the triangle is mechanically induced, while the circle is triggered by the magnetic field), and f) a comparison of the time required for phase transitions.

A quantitative analysis of the composites’ thermodynamic properties is essential for evaluating their thermal energy storage capabilities. Thus, we first used differential scanning calorimetry (DSC) to investigate the phase transition behavior of pure AMP and the AMP/NdFeB composites. As shown in **Figure** **5**a, pure AMP only undergoes an endothermic phase transition at ≈360 K (Δ*S* = 618.97 J kg^−1^ K^−1^, Δ*H* = 223.4 J g^−1^) similar to our previous report.^[^ ^30^ ^]^ For these AMP/NdFeB composites, the endothermic peaks become progressively sharper and shift ≈3.5 K to lower temperature with increasing NdFeB content, which suggests an enhancement in thermal transport. Δ*S* and Δ*H* drastically decrease to 143.6 J kg^−1^ K^−1^ and 51.2 J g^−1^ as the unit mass of the active AMP component is reduced and excessive NdFeB powder absorbs a part of heat (Figure 5b). We found that 20% AMP/NdFeB composites still displayed large ΔS (507.6 J kg^−1^ K^−1^) and Δ*H* (181.1 J g^−1^), unlike the others. These results suggest an optimal composite ratio exists for peak performance.

Therefore, infrared thermography provides an intuitive method for identifying the optimal composition by visualizing the macroscopic heat release performance of AMP/NdFeB composites, as shown in Figure 5c and S5, Supporting Information. First, a single‐point phase transition initiated by mechanical method (needle puncture) at a pure AMP (0.5 g, 2 × 2 cm^2^) sample, for instance, propagates slowly through the material, requiring almost 3 min for full crystallization and yielding a maximum temperature (*T*_max_) up to ≈345 K. In stark contrast, the magnetic field induces simultaneous, multipoint nucleation, which dramatically accelerates the overall heat release. These images clearly show that the propensity for multipoint nucleation is enhanced at higher mixing ratios of NdFeB, with identical sample area and mass. For example, the 20% and 40% AMP/NdFeB exhibit two distinct nucleation points spending over 1.5 min for full crystallization, while the 60% sample shows at least five points, leading to a much faster phase transition (less than 1.5 min). Critically, two‐point phase transition highest temperatures determined via magnetic method at 20% (347.5 K) and 40% (346.1 K) AMP/NdFeB concentrations exceed those from single‐point by a mechanical way. In summary, the advantage of this magnetic triggering approach becomes evident when compared to conventional mechanical methods.

To figure out the variation tendency between the mixed ratio and *κ*, we carried out transient plane source method (TPS) tests on two cylindrical crystal‐state samples. The DSC phase transition peaks of the mixed sample were sharp, which might be due to the improved thermal conductivity (*κ*) of the mixed NdFeB (5 ≈ 18 W m^−1^ K^−1^).^[^ ^43^, ^44^ ^]^ The improved thermal transport was quantitatively verified by measuring the *κ*, which is a measure of a material's capacity to conduct heat. Since direct measurement on the supercooled liquid is unfeasible due to contact‐induced crystallization, we measured the *κ* of cylindrical solid‐phase samples using the planar heat source method. Figure 5d reveals a clear positive correlation between the NdFeB content and thermal conductivity. Notably, the introduction of just 20% NdFeB enhances *κ* by ≈44.2%, from 0.52 W m^−1^ K^−1^ (pure AMP) to 0.75 W m^−1^ K^−1^ (20%). At an 80%, *κ* is prominently enhanced by over 200% to 1.61 W m^−1^ K^−1^. Besides, we observed the actual thermal conductivity of a cylindrical crystal‐state samples with the same thickness (5 mm) on a 70 °C hot table with an infrared camera, and the results further confirmed that the mixed sample obviously conducts higher temperatures in a shorter time as shown in Figure S4, Supporting Information.

In order to investigate Δ*T*_ad_ dependence on time clearly, Figure 5e shows the temperature change depending on time extracted from infrared temperature change recording, correlated with Figure 5d and Figure S5, Supporting Information. The high efficiency of heat release is also reflected in a higher Δ*T*_ad_, such as 20% and 60% of the samples achieving complete phase transition in 100 and 70 s, respectively (before the temperature variation starts to drop sharply), with higher temperature variations of ≈47.6 and ≈44.9 K. The thermal performance of 40% AMP/NdFeB is similar to that of 20%, but the temperature variation of the former one is slightly lower. However, 10% constitutes the critical lower bound for the mixing ratio, beyond which samples fail to undergo magnetic field‐induced phase transitions, as shown in Figure S6, Supporting Information. Moreover, a higher mixture ratio does not represent a better performance. Furthermore, Δ*T*_ad_ remains stable without degradation over five thermal cycles in air, confirming excellent cyclability (Figure S7, Supporting Information). To evaluate the efficiency of phase transitions induced by two methods, Figure 5f contrasts the time required for one‐point and multipoint phase transitions with different mixed ratios, related to Figure 5e. Compared to the pure sample, 20%, 40%, and 60% AMP/NdFeB reduce phase transition time by 50, 50, and 80 s, respectively, and increase efficiency from 33.3% to 53.3% as the ratio rises. Although 80% ratio sample is more likely to induce multipoint phase transition, its phase change rate is the slowest spending 120 s to finishing. Additionally, there are stability issues and Δ*T*_ad_ also shows a deteriorating phenomenon (as low as 33.8 K), as shown in Figure S5, Supporting Information. This is because under the same amount of AMP (constant heat), excessive NdFeB hinders the conduct of spontaneous phase transition and absorbs most of the heat which is consistent with its small Δ*S*. These initial findings revealed a critical tradeoff: increasing the NdFeB content enhances *κ*, which is beneficial for rapid heat transfer, but diminishes Δ*S*, which is detrimental to storage capacity. In conclusion, the 20% AMP/NdFeB has the best heat storage performance and can achieve a more stable and raised efficient heat release rate at a low mixing ratio.

## 3 Conclusion

This work addresses two long‐standing bottlenecks in solid‐state phase change thermal storage: the lack of noncontact control and intrinsically low thermal conductivity. By embedding magnetically responsive NdFeB particles into AMP plastic crystals, we demonstrate, for the first time, a magnetically triggered supercooled phase transition under an ultralow field (≈0.04 T), accompanied by substantially improved heat transfer. The dispersed NdFeB particles not only enable remote actuation but also facilitate simultaneous multipoint crystallization, thereby accelerating the heat‐release process. Nevertheless, these functionalities exhibit a competitive balance, as excessive magnetic loading diminishes the overall thermal effect due to parasitic heat absorption. We identify an optimal composition of 20% AMP/NdFeB, which delivers a rapid temperature rise of ≈47.6 K within 100 s, together with ≈44.2% higher thermal conductivity and ≈33.3% enhanced heat‐release efficiency compared to pristine AMP. These findings establish a design principle for magneto‐responsive plastic crystal systems and open a pathway toward high‐performance, programmable, and scalable thermal batteries.

## 4 Experimental Section

4.1

4.1.1

##### Preparation of the AMP/NdFeB Composites

Weigh and mix 0.5 g of AMP and NdFeB powder according to mass ratios of 0%, 20%, 40%, 60%, and 80% respectively, and then carry out mechanical grinding to make them evenly mixed. The mixture was then placed in a specific mold (2 × 2 cm^2^), heated, and then cooled down to transform it into a supercooled glassy state, which was used for subsequent characterization and performance measurement. Prepare cylindrical samples of AMP/NdFeB in a crystalline state with a diameter of 3 cm and a thickness of ≈5 mm through the mold for thermal conductivity testing.

##### X‐Ray Diffraction, Raman Spectroscopy, and Magnetic Property

X‐ray diffraction (XRD) patterns at 298 K were obtained at a diffractometer (SmartLab 3 kW, Rigaku) equipped with Cu‐Kα radiation (1.5406 Å). Temperature‐dependent Raman spectra were acquired at the high‐resolution Raman spectrometer (LabRAM HR Evolution, HORIBA Jobin Yvon) at 298 K. The sample was excited by radiation with a wavelength of 532 nm generated by a helium‐neon gas laser operating at 8.5 mW. The magnetic hysteresis loops *(M*‐*H* loops) of the AMP and AMP/NdFeB powder were measured by a SQUID magnetometer (Quantum Design, MPMS).

##### Strain Response to the Magnetic Field

Speckle patterns were prepared on different supercooled samples within a 2 × 2 cm^2^ glass mold. Elongated magnets were placed at the rear of the mold to apply magnetic fields ranging from 0.04 to 0.16 T, as measured by a TUNKIA TD8650 tesla meter. The influence of the magnetic field on the phase transition and the mechanical response of the mixed samples was investigated. The magnetic field‐induced phase transition was monitored in situ via strain evolution analysis using a DIC system equipped with a Basler camera.

##### Thermodynamic Performance

Heat flow data for AMP and AMP/NdFeB powder at ambient pressure were collected using a differential scanning calorimeter (NETZSCH DSC 200F3 for scans at 10 K min^−1^). The phase‐transition temperature was defined as the temperature at which the heat flow peaked. The entropy changes under constant pressures were calculated by integrating the heat flow *Q*(*T*), after subtracting the baseline, in the temperature interval between *T*_1_ and *T*_2_.

$$\Delta S=\int_{T_{1}}^{T_{2}}\frac{1}{T}\frac{Q(T)}{T\prime }dT$$

where *T*′ is the temperature ramping rate.^[^ ^15^, ^45^ ^]^ We directly measured the temperature change of AMP during a pressure‐induced phase transition by means of thermography. A long‐shaped magnets are respectively used to afford the magnetic field to induce single‐point and multipoint phase transitions of supercooled AMP/NdFeB samples. A needle was used to apply pressure to a specific position for supercooled AMP/NdFeB composites. The temperature evolution of the sample during the pressure‐induced phase transition was recorded using an infrared camera (Fluke TiX580). Two cylindrical samples of all AMP/NdFeB composites were respectively prepared using a 3 cm diameter mold for testing thermal conductivity. The thermal conductivity of each sample was measured using the Hotdisk 2500S via TPS. After discarding the initial measurement, the average of the subsequent three measurements was calculated as the final value.

## Supporting Information

Supporting Information is available from the Wiley Online Library or from the author.

## Conflict of Interest

The authors declare no conflict of interest.

## Supporting information

Supplementary Material

## Data Availability

The data that support the findings of this study are available from the corresponding author upon reasonable request.
