# Supplementary material for: High‐Efficiency Thermal Battery Regulated by Ultralow Magnetic Fields
Source: Small Sci. 2025 Dec 12;6(1):e202500498. doi: 10.1002/smsc.202500498 (PMC12798784; doi:10.1002/smsc.202500498)
Supplement: Supplementary file 1 — Supplementary Material [file SMSC-6-e202500498-s001.pdf]

Supporting Information

High-Efficiency Thermal Battery Regulated by Ultra-Low Magnetic Fields

Lingli Li, Haoyu Wang, Zhiyu Wang, Dan Huang, Kun Zhang, Bing Li**

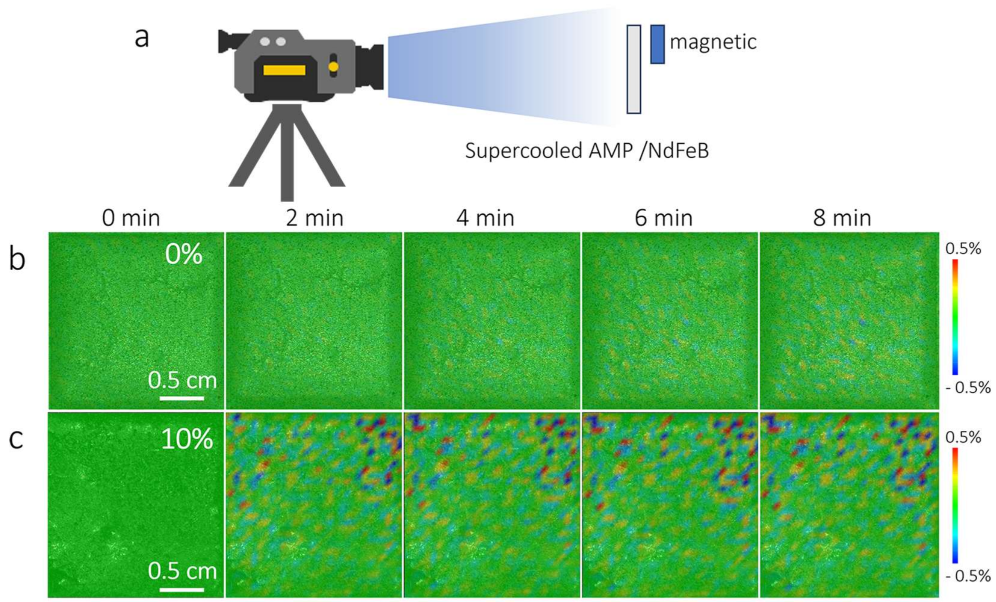

Figure S1. **Strain distribution induced by magnetic field.** (a) schematic diagram for DIC tests, (b) 0 % did not undergo any deformation in response to the magnetic field, (c) 10 % showed obvious strain change (~0.5%) in the area affected by the magnetic field. It is suggested that the magnetically controlled phase transition of AMP is an indirect response resulting from the incorporation of NdFeB powder.

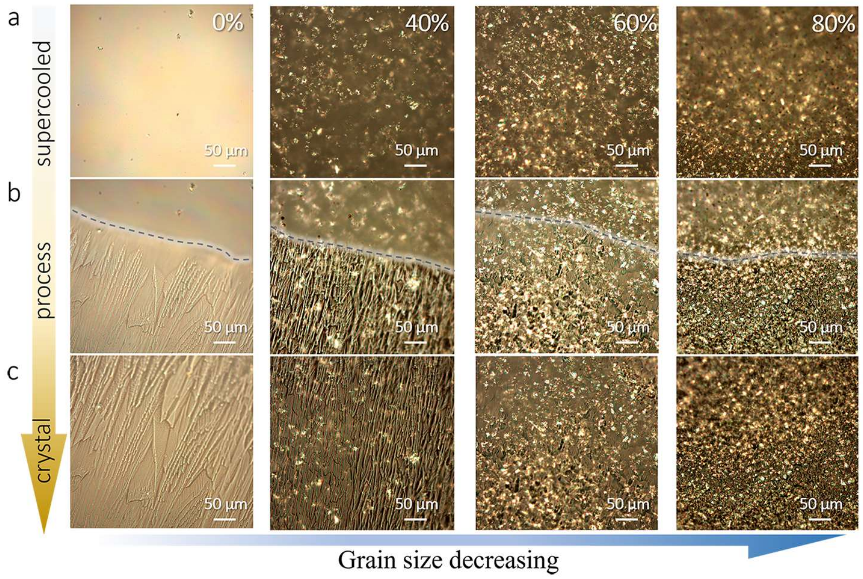

Figure S2. The optical photo of 0%, 40%, 60% and 80% AMP/NdFeB crystallizing processing. (a) supercooled state, (b) phase transition process, (c) crystal state. Supercooled states with different mixing ratios all exhibit amorphous morphology until they crystallize, forming crystal phases with distinct grain boundaries. Meanwhile, the grain size decreases as the mixing ratio increases, because the increased number of particles provides abundant nucleation sites.

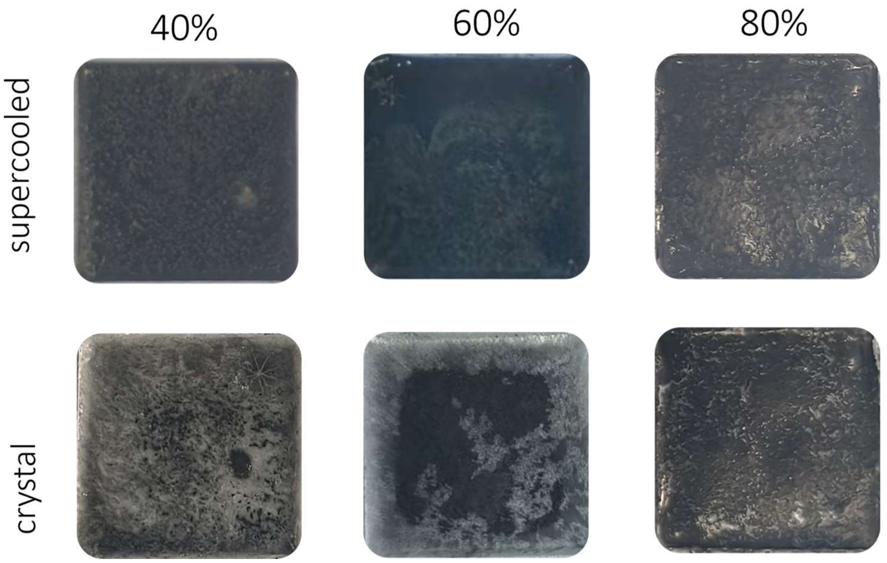

Figure S3. Optical photos of 40%, 60% and 80% AMP/NdFeB at supercooled and crystal states respectively. The crystal states emit a white color.

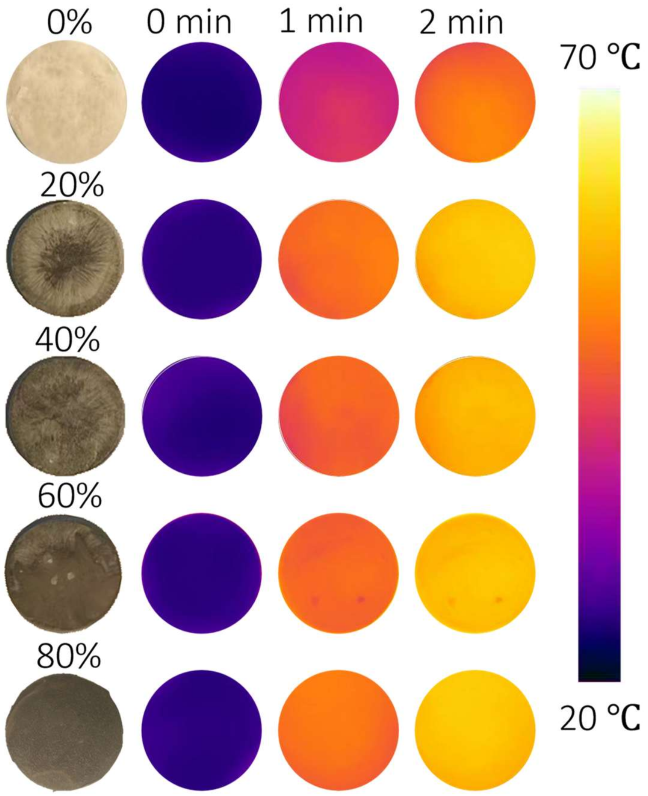

Figure S4. In situ detection of surface temperature of AMP and AMP/NdFeB composites with same thickness (5 mm) recorded by an infrared thermal imager at 70 ℃ hot plate. All five composites reached significantly higher temperatures than the pure AMP one minute faster.

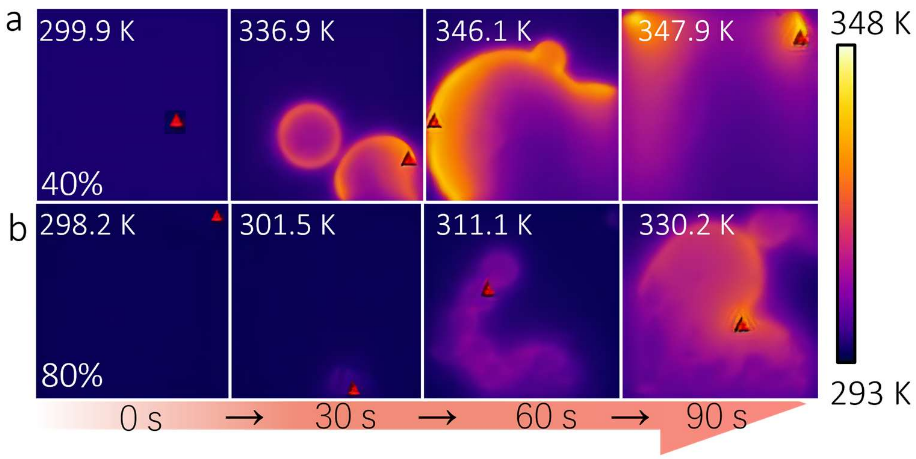

Figure S5. Infrared thermography pictures exhibiting the heat releasing processes of multi-point phase transition. (a) 40% AMP/NdFeB shows three-points phase transition and ΔT approaching to 47 K, (b) 80% AMP/NdFeB exhibits more points begin to undergo phase transition, while the thermal releasing performance is the worst.

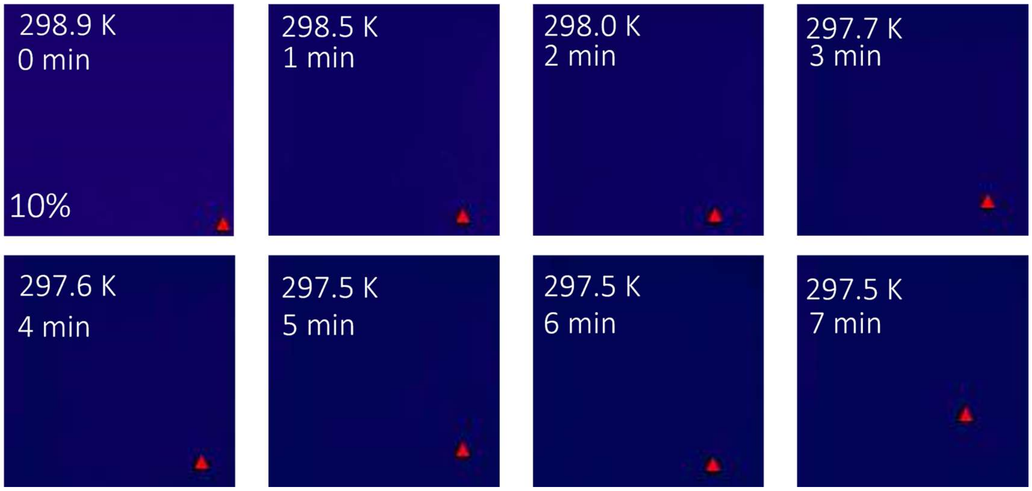

Figure S6. Infrared thermography pictures shows that a small magnetic field is hard to trigger the phase change in 10% AMP/NdFeB, because of the low content of NdFeB powder.

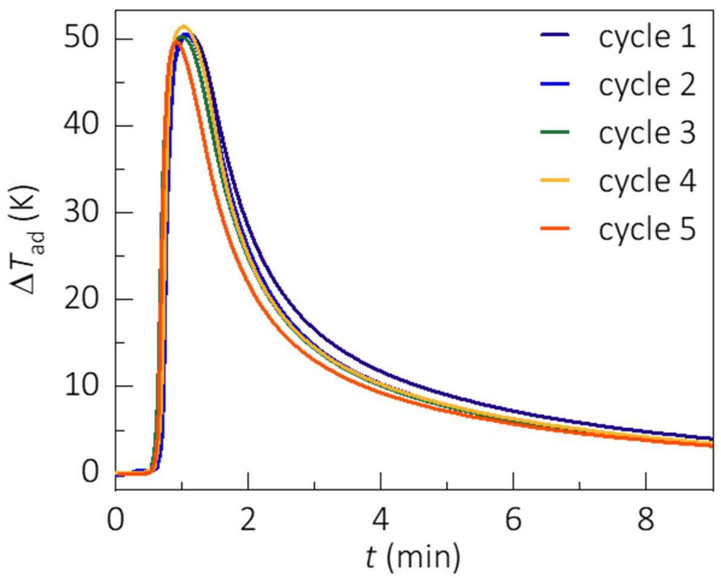

Figure S7. Thermal cycles of the supercooled optimal 20% AMP/NdFeB composite. The maximum temperature change approaches ~50 K and shows no degradation after 5 cycles, indicating excellent cyclability.
